# Quantum electric-dipole liquid on a triangular lattice

**DOI:** 10.1038/ncomms10569

**Published:** 2016-02-04

**Authors:** Shi-Peng Shen, Jia-Chuan Wu, Jun-Da Song, Xue-Feng Sun, Yi-Feng Yang, Yi-Sheng Chai, Da-Shan Shang, Shou-Guo Wang, James F. Scott, Young Sun

**Affiliations:** 1 Beijing National Laboratory for Condensed Matter Physics, Institute of Physics, Chinese Academy of Sciences, Beijing 100190, China; 2 Hefei National Laboratory for Physical Sciences at the Microscale, University of Science and Technology of China, Hefei 230026, China; 3 Key Laboratory of Strongly-Coupled Quantum Matter Physics, Chinese Academy of Sciences, Hefei, Anhui 230026, China; 4 Collaborative Innovation Center of Advanced Microstructures, Nanjing, Jiangsu 210093, China; 5 Cavendish Laboratory, University of Cambridge, J. J. Thomson Avenue, Cambridge CB3 0HE, UK; 6 Schools of Chemistry and Physics, St. Andrews University, St. Andrews, Fife, Scotland KY16 9ST, UK

## Abstract

Geometric frustration and quantum fluctuations may prohibit the formation of long-range ordering even at the lowest temperature, and therefore liquid-like ground states could be expected. A good example is the quantum spin liquid in frustrated magnets. Geometric frustration and quantum fluctuations can happen beyond magnetic systems. Here we propose that quantum electric-dipole liquids, analogues of quantum spin liquids, could emerge in frustrated dielectrics where antiferroelectrically coupled electric dipoles reside on a triangular lattice. The quantum paraelectric hexaferrite BaFe_12_O_19_ with geometric frustration represents a promising candidate for the proposed electric-dipole liquid. We present a series of experimental lines of evidence, including dielectric permittivity, heat capacity and thermal conductivity measured down to 66 mK, to reveal the existence of an unusual liquid-like quantum phase in BaFe_12_O_19_, characterized by itinerant low-energy excitations with a small gap. The possible quantum liquids of electric dipoles in frustrated dielectrics open up a fresh playground for fundamental physics.

## Abstract (web summary)

In magnetic materials, geometry-defined competing interactions between spins combined with quantum fluctuations can present the possibility of quantum liquid states which do not order even as 0K is approached. Here, the authors present an analogue built from electric dipoles on a triangular lattice.

## Graphical abstract

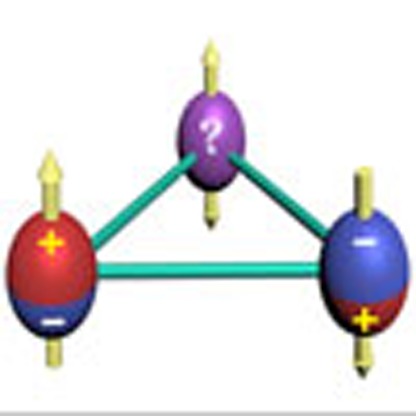

Geometric frustration arises on various triangle-based lattices such as one-dimensional (1D) trestle lattice, two-dimensional (2D) triangular and kagome lattices and three-dimensional (3D) B-site spinel and pyrochlore lattices, and are typically investigated in spin systems^1^,^2^,^3^. It has become well known that the introduction of quantum fluctuations in geometrically frustrated magnets gives rise to a rich variety of interesting quantum phases^3^,^4^,^5^, as discussed with both the transverse Ising models^6^,^7^,^8^,^9^ and Heisenberg models^10^,^11^. Especially, exotic quantum spin liquids (QSLs), characterized by either gapped or gapless itinerant excitations^3^,^4^, have been theoretically predicted to show extremely intriguing phenomena. Compared with the impressive progress and diversity in theory, nevertheless, a clear identification of QSLs in real materials has proved challenging, with a very limited number of candidates reported so far^12^,^13^,^14^,^15^,^16^.

Similar to the situation of spin lattices in magnets, geometric frustration can occur in lattices made of electric dipoles in dielectrics. In the case of small electric dipoles with significant quantum fluctuations persisting down to *T*=0 K, exotic disordered quantum phases, such as a quantum electric-dipole liquid (QEL), could emerge in certain conditions. In fact, some theoretical models proposed for ultracold dipolar particles trapped on 2D frustrated optical lattices have predicted topological quantum phases with fractional excitations^17^. In a QEL, the electric dipoles are highly entangled with one another in a form of quantum dimers (pairs of antiparallel dipoles) and continue to fluctuate in the resonating valence bond state, a picture qualitatively similar to a QSL. However, we must emphasize that the QEL should have distinctive features from QSLs because electric dipole and spin have important differences^18^. For instance, electric dipole neither has intrinsic angular momentum nor exhibits quantum precession as magnetic dipole (spin) does. Moreover, the nature of short- and long-range interactions between electric dipoles is very different from that of spins^19^. This could lead to a very different phase diagram between QSLs and QELs.

Frustration in dielectrics has been previously studied in materials with competing ferroelectric (FE) and antiferroelectric (AFE) constituents such as the KH_2_PO_4_/NH_4_H_2_PO_4_ (KDP-ADP) family or containing random-site impurities such as KTaO_3_:Li, which usually result in electric-dipole glasses similar to spin glasses^20^,^21^. However, the geometric origin of frustration and cooperative liquid-like quantum phases has been largely ignored in the studies of dielectrics. On the other hand, the role of quantum fluctuations in dielectrics has been noticed since 1970s when people were studying the abnormal dielectric behaviour of SrTiO_3_ (ref. 22). It was proposed that quantum fluctuations in SrTiO_3_ prevent the onset of long-range FE order so that a quantum paraelectric state persists down to zero temperature^23^. Since then, quantum paraelectricity has been reported in a number of perovskite oxides with similar structures to SrTiO_3_, such as CaTiO_3_, EuTiO_3_ and KTaO_3_. The quantum paraelectrics provide a new playground for the study of quantum critical phenomena^18^; however, it seems hopeless to search for the QELs in those perovskite quantum paraelectrics because their crystalline structures and FE interactions usually do not introduce geometric frustration.

In this work, we demonstrate that both geometric frustration and strong quantum fluctuations can be simultaneously achieved in a unique quantum paraelectric hexaferrite BaFe_12_O_19_ in which small electric dipoles that originated from the off-centre displacement of Fe^3+^ in the FeO_5_ bipyramids constitute a 2D triangular lattice. Our experiments including dielectric permittivity, heat capacity and thermal conductivity measured down to 66 mK suggest the existence of a very unusual liquid-like ground state, characterized by itinerant low-energy excitations with a small gap. We consider this nontrivial quantum phase as a possible candidate of the QELs. The quantum liquids of electric dipoles in frustrated dielectrics provide a new playground for fundamental physics and may find applications in quantum information and computation as well.

## Result

### Geometric frustration in BaFe_12_O_19_

**Figure 1:**
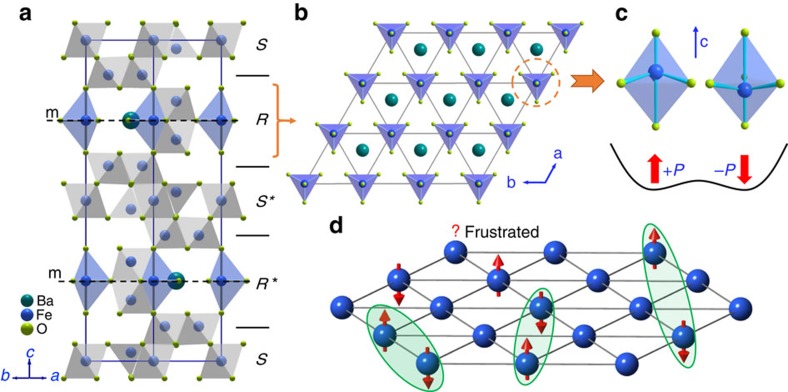
Uniaxial electric dipoles on a triangular lattice in BaFe_12_O_19_. (**a**) Crystal structure of the M-type hexaferrite BaFe_12_O_19_. It consists of alternate stacks of *S* and *R* blocks along the *c* axis. The asterisk symbols indicate that the corresponding blocks rotate about the *c* axis by 180°. The Fe^3+^ ions occupy three different sites: octahedral, tetrahedral and bipyramidal (blue) sites. A mirror plane (m) bisects equally the bipyramids in the R/R^*^ blocks. (**b**) The 2D triangular lattice of FeO_5_ bipyramids in each *R*/R* block. (**c**) Illustration of Fe^3+^ off-equator displacements in the FeO_5_ bypyramid. The upward or downward displacements at two 4*e* sites give rise to small electric dipoles along the *c* axis. Quantum fluctuations between two 4*e* sites persist to *T*=0 K. (**d**) Frustrated electric dipoles on a triangular lattice. Each site contains an Ising-type electric dipole (red arrow), while the neighbouring interactions favour anti-alignment. Quantum dimers (marked by green ovals) with either short-range or long-range entanglement continue to fluctuate and result in a QEL.

Recently, we have discovered that the M-type hexaferrites, such as BaFe_12_O_19_, belong to a completely new family of quantum paraelectrics^24^. Other hexaferrites containing the FeO_5_ bipyramids in their crystal structures, such as the W-, Z-, X- and U-type hexaferrites, are also likely candidates of quantum paraelectrics^24^. The M-type hexaferrite BaFe_12_O_19_ is one of the most popular magnetic materials with a wide use in magnetic credit cards, bar codes, small motors and low-loss microwave devices^25^ because of its superior properties of ferrimagnetic ordering with a strong ferromagnetic moment and a very high Néel temperature (∼720 K), high resistivity, as well as low cost of synthesis. The crystal structure of BaFe_12_O_19_ is shown in Fig. 1a. It can be described by a periodically stacking sequence of two basic building blocks—*S* block and *R* block along the *c* axis. The Fe^3+^ ions occupy three different kinds of sites: octahedral, tetrahedral and bipyramidal sites. In particular, the FeO_5_ bipyramids only exist in the middle of the *R*/R^*^ blocks and form a triangular lattice in the *ab* plane (Fig. 1b).

Previous experiments including Mössbauer spectroscopy^26^, X-ray diffraction^27^ and neutron diffraction^28^ have revealed the existence of off-equatorial displacements for Fe^3+^ at Wyckoff position of 2b site inside the FeO_5_ bipyramids to minimize the total energy, which results in two adjacent Wyckoff positions of 4e sites with a lowered symmetry (Fig. 1c). The off-equatorial displacement (4e–4e distances are 0.176(5) Å at 4.2 K and 0.369(5) Å at room temperature)^26^ would induce a small local electric dipole *P* along the *c* axis in each FeO_5_ bipyramid (Fig. 1c). A dynamic displacement persists down to the lowest temperature because of the significant quantum tunnelling between two 4e sites and the weak dipole–dipole coupling along the *c* axis. Consequently, a quantum paraelectric behaviour without long-range electric ordering has been observed in BaFe_12_O_19_ (ref. 24).

More importantly, these electric dipoles associated with the FeO_5_ bipyramids reside on a triangular lattice in each *R*/R^*^ block. Because the *R*/R^*^ blocks are well separated by the *S*/S* blocks, this triangular lattice thus has a 2D feature. Consequently, a dielectric system with uniaxial (Ising-type) electric dipoles on a 2D triangular lattice is practically achieved in BaFe_12_O_19_ (Fig. 1c). If the neighbouring dipole–dipole interaction favours anti-alignment, the system confronts frustration and has a very large degeneracy of ground states. In this sense, BaFe_12_O_19_ is a very unique quantum paraelectric system compared with those previously known perovskite quantum paraelectrics: first, it has uniaxial electric dipoles, whereas perovskite SrTiO_3_ is pseudocubic with multiple easy axis; second, it experiences geometrical frustration, whereas there is no evidence of geometrical frustration in SrTiO_3_ and other perovskite quantum paraelectrics. Thus, the quantum paraelectric BaFe_12_O_19_ sets up a promising candidate to search the proposed QELs, where an assembly of quantum dimers (pairs of dipoles) with long-range entanglement continues to fluctuate (Fig. 1d). We then employ a series of experimental techniques to resolve the ground state of BaFe_12_O_19_.

### Dielectric permittivity

**Figure 2:**
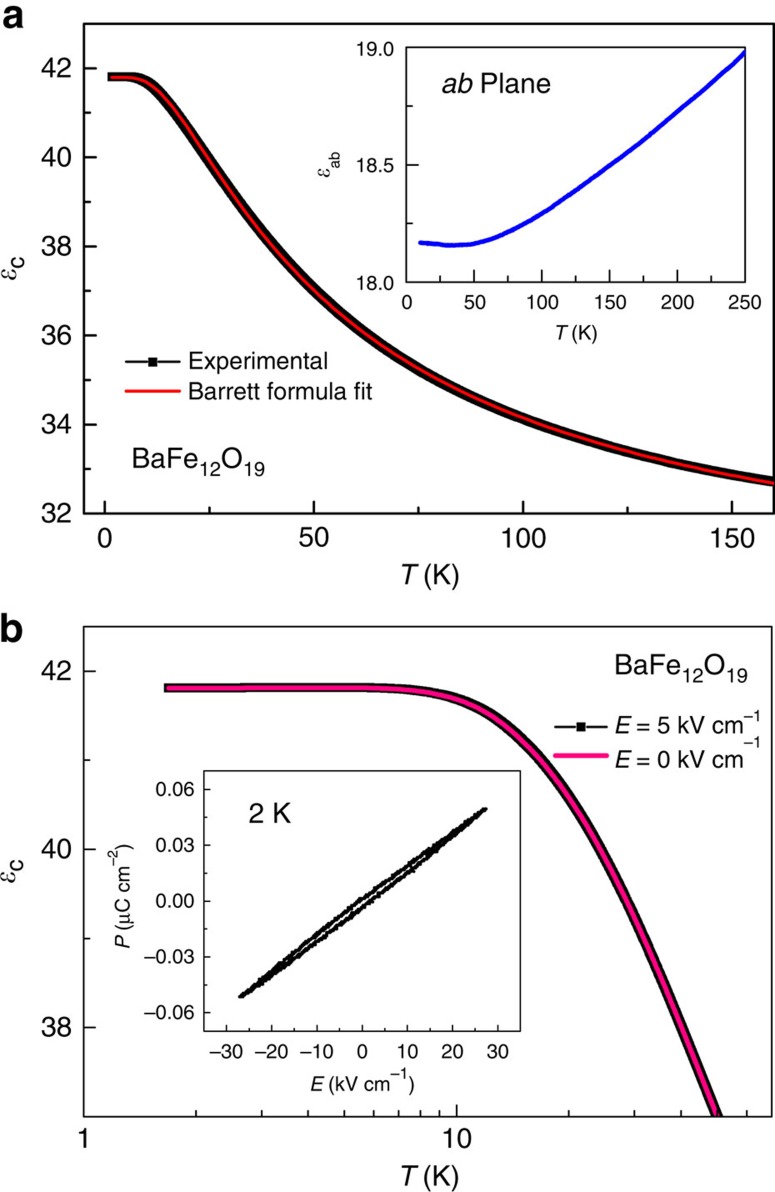
Dielectric permittivity of BaFe_12_O_19_. (**a**) The temperature dependence of *c* axis dielectric permittivity *ɛ*_*c*_. The red solid line is the fitting curve to the Barrett formula. The negative fitting parameter *T*_0_=−22.9(1) K suggests the AFE interaction. The inset shows the dielectric permittivity measured along the [100] direction in the *ab* plane. (**b**) The temperature dependence of dielectric permittivity *ɛ*_*c*_ measured with d.c. bias electric fields. A bias electric field of 5 KV cm^−1^ has no detectable influence on the quantum paraelectric behaviour. The inset shows the *P*–*E* loop at 2 K. The nearly linear response and the low values of *P* imply the AFE coupling.

A prerequisite of a QEL is the AFE interaction between neighbouring dipoles. To confirm the AFE coupling in BaFe_12_O_19_, we have made a careful analysis on the low-temperature dielectric permittivity. As shown in Fig. 2a, the dielectric permittivity along the *c* axis (*ɛ*_*c*_) of BaFe_12_O_19_ increases steadily with decreasing temperature but remains nearly constant below ∼5.5 K. No dielectric phase transition is observed down to 1.5 K. This dielectric behaviour evidences a quantum paraelectricity, similar to that in SrTiO_3_. The quantum paraelectric behaviour can be well described by the mean-field Barrett formula^29^:


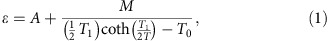


where *A* is a constant, *T*_0_ is proportional to the effective dipole–dipole coupling constant and the positive and negative values correspond to FE and AFE interactions, respectively. *T*_1_ represents the tunnelling integral and is a dividing temperature between the low-temperature region where quantum fluctuation is important and the high-temperature region where quantum effect is negligible. *M=nμ*^*2*^*/k*_B_, where *n* is the density of dipoles, *μ* denotes the local dipolar moment and *k*_B_ is the Boltzmann constant.

After fitting the *ɛ*_*c*_ below 160 K to the Barrett formula, we obtained *T*_0_=−22.9(1) K and *T*_1_=47.3(1) K. The negative *T*_0_ confirms the AFE coupling between electric dipoles. We note that recent first-principle calculations^30^ also predicted the AFE interaction with frustration in BaFe_12_O_19_. The relative strength of quantum fluctuations can be estimated by ∼|*T*_1_/*T*_0_|=2.06, which is likely high enough to favour a liquid ground state rather than an ordered or glass phase. The uniaxial anisotropy is evidenced by comparing the dielectric permittivity along the *c* axis with that in the *ab* plane. As seen in the inset of Fig. 2a, the in-plane *ɛ* decreases slowly with decreasing temperature (less than 1 for a temperature interval of 250 K). The absence of a paraelectric behaviour in the *ab* plane is consistent with the uniaxial electric dipoles along the *c* axis.

Further lines of evidence of the AFE coupling in BaFe_12_O_19_ are presented in Fig. 2b. For those perovskite quantum paraelectrics with FE coupling, such as SrTiO_3_, a moderated electric field is able to drive the quantum paraelectric state into a long-range ordered FE state^31^. In strong contrast, for BaFe_12_O_19_, an external electric field of 5 KV cm^−1^ applied along the *c* axis has no detectable influence on the dielectric permittivity. This inertness to external electric fields may indicate the AFE interaction in BaFe_12_O_19_. Moreover, the *P–E* loop at 2 K (the inset of Fig. 2b) shows a nearly linear response with quite small polarization up to a high electric field of 30 kV cm^−1^, further implying the AFE coupling. It should be clarified that the magnetic moments of Fe^3+^ at the bipyramidal sites are all parallel along the *c* axis in the R/R* blocks (see Supplementary Fig. 1) so that there is no magnetic frustration on the triangular lattice.

### Heat capacity and thermal conductivity

The thermodynamic studies at temperatures as low as possible are crucial to identify the conjectured quantum liquid state, as they provide the key information on the spectrum of low-energy elementary excitations. Heat capacity and thermal transport measurements can probe the low-energy density of states as well as determine whether these low-energy excitations are localized or itinerant, and have been indispensably employed in the study of QSLs^32^,^33^,^34^.

**Figure 3:**
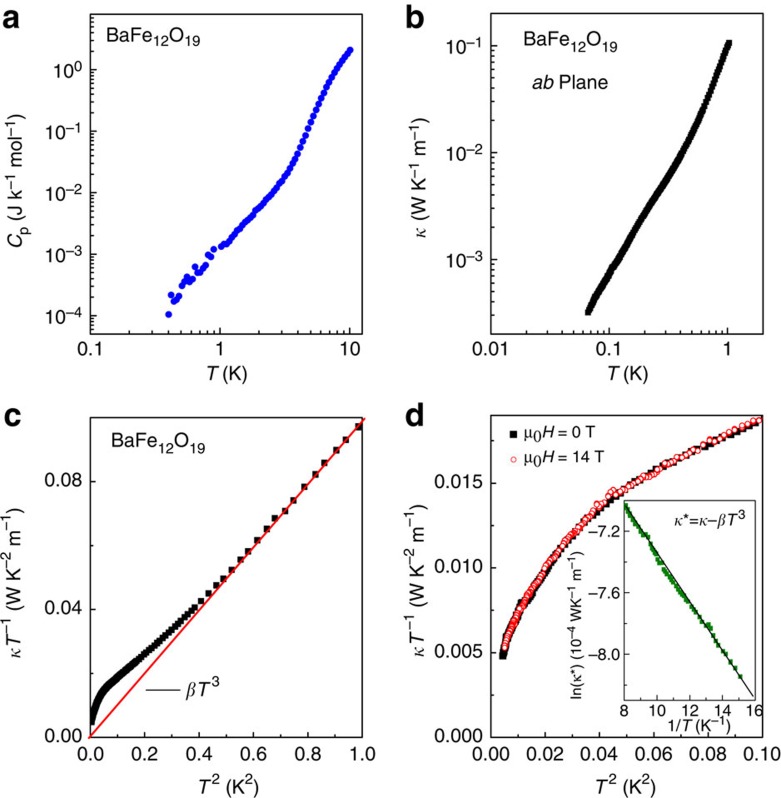
Heat capacity and thermal conductivity of BaFe_12_O_19_. (**a**) Heat capacity *C*_p_ as a function of temperature. No phase transition is detected down to 0.4 K. (**b**) Thermal conductivity *κ* measured in the *ab* plane as a function of temperature. No anomaly due to a phase transition is observed down to 66 mK. (**c**) The *ab* plane thermal conductivity divided by temperature plotted as a function of *T*^2^ below ∼1 K. The red solid line represents the expected thermal conductivity of phonons, *κ*=*βT*^3^, with *β*=0.098 WK^−4 ^m^−1^. Apparently, there is an extra contribution beside the phonon thermal conductivity below ∼650 mK. (**d**) The *κT*^−1^ versus *T*^2^ plot in the lowest temperature region. *κ*/*T* tends to vanish at the *T*→0 K limit. An applied magnetic field of 14 T along the *c* axis has no influence on the *ab* plane thermal conductivity in this low-temperature region. The inset shows the Arrhenius plot of κ*=*κ*−*βT*^3^ below ∼125 mK. The good linearity suggests gapped excitations with a small gap ∼0.16 K.

Since BaFe_12_O_19_ is a good insulator (see Supplementary Fig. 2) with long-range collinear ferrimagnetic ordering (*T*_N_=720 K), both the electronic and magnon contributions to the thermal dynamics become negligible at very low temperatures^35^. Therefore, its thermodynamics at low enough temperatures should be dominated by the lattice contribution only, and the well-known *T*^3^ dependence would be expected for both the heat capacity and thermal conductivity. Figure 3a shows the heat capacity (*C*_P_) of BaFe_12_O_19_ at low temperatures (*T*<10 K). No sharp anomaly due to a phase transition could be detected down to 0.4 K, in accordance with the quantum paraelectric behaviour. Unfortunately, the heat capacity data become scattered and noisy below ∼1 K, possibly because of the very small values that reach the resolution limit of our equipment. Thus, a quantitative analysis of the heat capacity data is not possible.

The thermal conductivity provides more reliable and critical information on the low-lying elementary excitations because it is sensitive exclusively to itinerant excitations and is totally insensitive to localized entities that may cause the nuclear Schottky contribution and plague the heat capacity measurements at low temperatures^33^,^34^. For example, although the heat capacity study^32^ suggested a gapless QSL in the frustrated triangular magnet *κ*-(BEDT-TTF)_2_Cu_2_(CN)_3_, the thermal conductivity measurements^33^ carried out down to 80 mK clarified instead a gapped QSL in the same material. We then have devoted a great effort to measure precisely the thermal conductivity of BaFe_12_O_19_ down to 66 mK.

Figure 3b shows the thermal conductivity *κ* measured in the *ab* plane as a function of temperature below ∼1 K. *κ* decreases rapidly with cooling, with a change more than 2 orders from 1 to 100 mK. No anomaly due to a phase transition is observed down to 66 mK. The thermal conductivity divided by temperature as a function of *T*^2^ is plotted in Fig. 3c. The data between 0.65 and 1 K exactly follow a linear relation with an extrapolation to the origin, in a good agreement with what expected for the phonon thermal conductivity, *κ*=*βT*^3^, with *β*=0.098 WK^−4 ^m^−1^. Nevertheless, there is apparently an extra contribution below ∼650 mK in addition to the normal phonon term, strongly suggesting the existence of abundant itinerant low-energy excitations other than phonons. Moreover, the thermal transport behaviour at the zero temperature limit provides the key information on the nature of these low-lying excitations. As seen in Fig. 3d, *κT*^−1^ in the *T*→0 K limit tends to vanish rather than having a finite residual value, immediately implying the absence of gapless excitations. Instead, the data at the lowest temperature regime (*T*<125 mK) can be fitted to


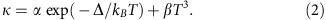


The inset of Fig. 3 shows an Arrhenius plot of κ^*^=*κ−βT*^3^ in the lowest temperature region. The good linearity confirms the validity of equation (2). The best fit gives *Δ*=0.16(1) K, which is much smaller than the effective dipole–dipole interaction constant *T*_0_ (∼23 K). The exponential behaviour of thermal conductivity at the zero temperature limit is very similar to that observed in the frustrated triangular magnet *κ*-(BEDT-TTF)_2_Cu_2_(CN)_3_, where a QSL with gapped excitations (*Δ*=0.46 K) was identified^32^. Therefore, the thermal transport behaviour excludes a frozen dipole glass or a classical paraelectric phase but strongly suggests an exotic liquid-like ground state.

To exclude the possibility that these itinerant low-lying excitations may have a magnetic origin, we further studied the influence of magnetic field on the thermal conductivity behaviour. As seen in Fig. 3d, a high magnetic field of 14 T applied along the easy *c* axis has no influence on the in-plane thermal conductivity in the lowest temperature range. The inertness of these low-lying excitations to external magnetic fields supports our argument that they stem from electric dipoles rather than spins.

## Discussion

We think that the above experimental observations, especially the thermal conductivity at the zero temperature limit, strongly indicate the existence of a nontrivial ground state of electric dipoles in BaFe_12_O_19_ that is a very unique dielectric system because both geometrical frustration and strong quantum fluctuations play an important role, a situation similar to frustrated antiferromagnets where people are looking for QSLs. This unusual ground state is characterized by itinerant low-energy excitations with a tiny gap (*Δ*=0.16 K) that is 140 times smaller than the effective dipole–dipole interaction constant *T*_0_ (∼23 K), a feature against a dipole glass or a classical dipole liquid. Thus, we consider it as a possible candidate of an exotic QEL. As current theoretical models proposed for frustrated spin systems, such as the transverse Ising models on a triangular lattice, are inadequate for the frustrated electric dipoles because the long-range dipole–dipole interactions are quite different from the short-range spin exchange interactions, a quantitative comparison between experiments and theories is not available at this stage. We expect that our experimental findings will stimulate theoretical efforts towards this interesting subject in the future. The present work serves as a start point to draw attention on frustrated quantum electric dipoles in dielectric materials where an abundance of exotic phenomena could be awaiting ahead.

## Methods

### Sample preparation

The single-crystal samples of BaFe_12_O_19_ were prepared with the flux method and were characterized with X-ray diffraction, as shown in Supplementary Fig. 3. Powders of BaCO_3_, Fe_2_O_3_ and fluxing agent Na_2_CO_3_ were weighed to a molar ratio of 1:1:1, and then were mixed and well ground. The ground raw powder was put in a Pt crucible and heated to 1,250 °C for 24 h in the air, and then cooled down to 1,100 °C at a rate of 3 °C min^−1^ and finally quenched to room temperature.

### Dielectric measurements

The dielectric measurements were carried out in a Cryogen-free Superconducting Magnet System (Oxford Instruments, TeslatronPT) down to 1.5 K. To measure the dielectric permittivity, silver paste was painted on the surfaces of a thin plate of crystal and annealed at 150 °C for ∼30 min to make good electrodes. An Agilent 4980A LCR meter was used to measure the dielectric permittivity with the frequency *f*=1 MHz.

### Heat capacity and thermal transport measurements

Heat capacity measurements were performed down to 0.4 K in a commercial Physical Properties Measurement System (Quantum Design) using a ^3^He refrigerator. A thin-plate-shaped sample with mass of 12.7 mg was used for this measurement. The contribution of attendant was measured separately and subtracted from the raw data. Thermal conductivity was measured between 60 mK and 1 K using the conventional steady-state ‘one heater, two thermometers' technique in a ^3^He–^4^He dilution refrigerator^36^,^37^. A parallelepiped-shaped sample with size of 2.0 × 0.57 × 0.11 mm^3^ was cut from the as-grown crystals for thermal conductivity measurements. A chip heater and two RuO_2_ chip sensors are attached to the sample with gold wires. The temperature difference between the two thermometers was controlled to be typically 3% of the sample temperature. To minimize heat leak, superconducting NbTi wires with 15 μm diameter are used as the leads of the chip sensors.

## Additional information

**How to cite this article:** Shen, S.-P. *et al*. Quantum electric-dipole liquid on a triangular lattice. *Nat. Commun.* 7:10569 doi: 10.1038/ncomms10569 (2016).

## Supplementary Material

Supplementary Information Supplementary Figures 1-3
