# Supplementary material for: Quantum electric-dipole liquid on a triangular lattice
Source: Nat Commun. 2016 Feb 4;7:10569. doi: 10.1038/ncomms10569 (PMC4743005; doi:10.1038/ncomms10569)
Supplement: Supplementary Information — Supplementary Figures 1-3 [file ncomms10569-s1.pdf]

Supplementary Information

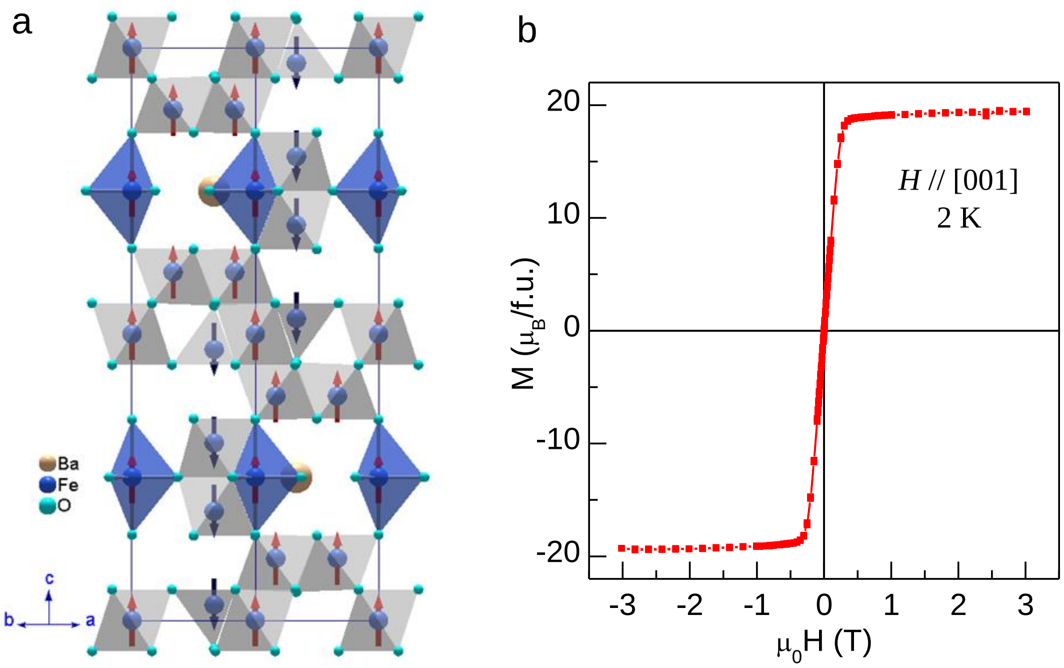

Supplementary Fig. 1 (a) Magnetic structure of $BaFe_{12}O_{19}$. The magnetic moments at the FeO_5 bipyramidal sites are parallel to each other so that there is no magnetic frustration on the triangular lattice. (b) The *M*-*H* hysteresis curve of $BaFe_{12}O_{19}$ measured at 2 K along *c* axis. It is consistent with a long-range collinear ferrimagnetic ordering.

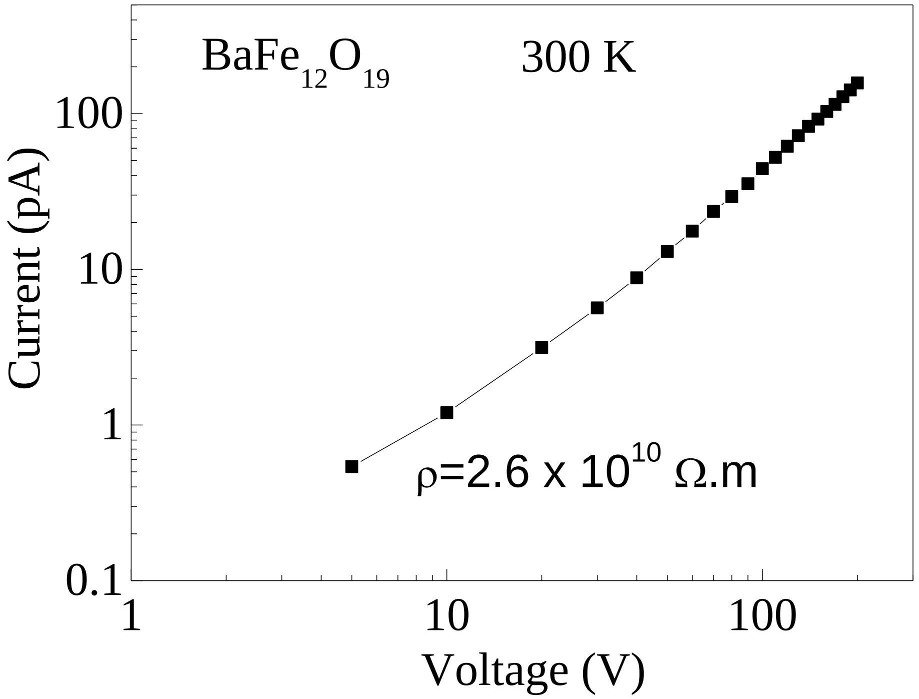

Supplementary Fig. 2 The *I-V* characteristic along c axis of $BaFe_{12}O_{19}$ measured at room temperature. The sample is highly insulating even at room temperature with a resistivity more than 10^{10} Ω.m, suggesting a good quality of the grown single crystals.

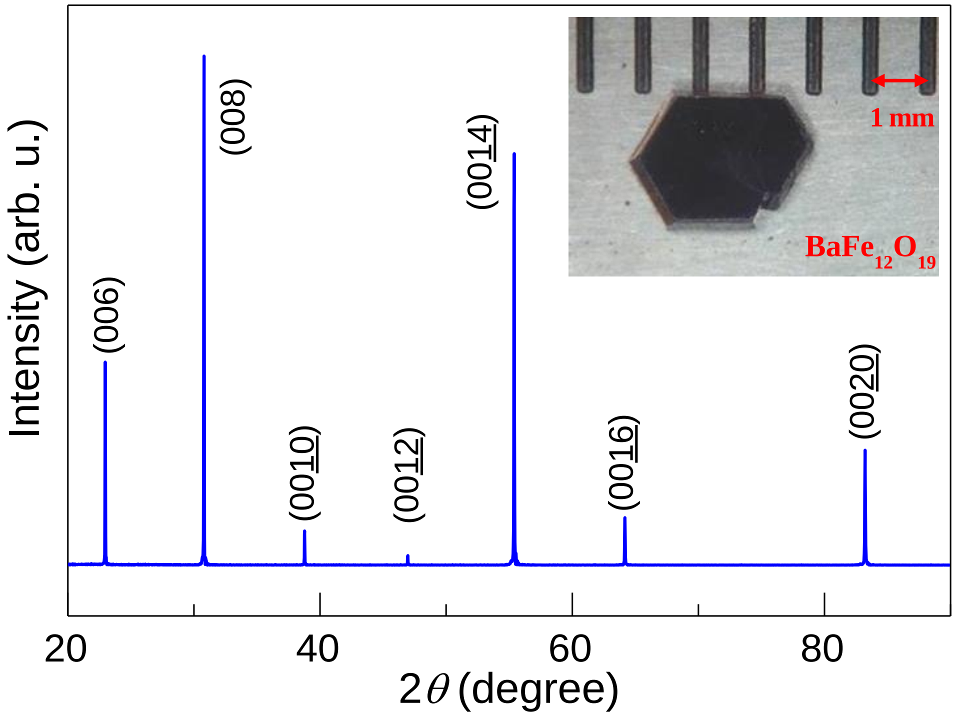

Supplementary Fig. 3 The single-crystal x-ray diffraction pattern of $BaFe_{12}O_{19}$ at room temperature. The inset shows a picture of flux-grown single crystals.
